# Three New Oblongolides from *Phomopsis* sp. XZ-01, an Endophytic Fungus from *Camptotheca acuminate*

**DOI:** 10.3390/molecules16043351

**Published:** 2011-04-19

**Authors:** Ting Lin, Guang Hui Wang, Xiang Lin, Zhi Yu Hu, Quan Cheng Chen, Yang Xu, Xiao Kun Zhang, Hai Feng Chen

**Affiliations:** 1 School of Pharmaceutical Sciences, Xiamen University, 422 South Siming Road, Xiamen, Fujian 361005, China; E-Mail: guanghui@xmu.edu.cn (G.H.W.); chenqc@xmu.edu.cn (Q.C.C.); xu_yang@xmu.edu.cn (Y.X.); xzhang@burnham.org (X.K.Z.); 2 Institute of Life Sciences, Fuzhou University, No.2 Xueyuan Rd., University Town, Fuzhou, Fujian 350108, China; E-Mail: linx1981@gmail.com (X.L.); 3 School of Life Sciences, Xiamen University, Fujian 361005, China; E-Mail: huzhiyu@xmu.edu.cn (Z.Y.H.)

**Keywords:** *Camptotheca acuminate*, endophytic fungus, *Phomopsis* sp. XZ-01, oblongolides, new metabolites

## Abstract

Four new metabolites, including three new oblongolides named C1, P1, and X1 (**1**-**3**) and 6-hydroxyphomodiol (**10**), along with eight known compounds – oblongolides B (**4**), C (**5**), D (**6**), O (**7**), P (**8**) and U (**9**), (3*R*,4a*R*,5*S*,6*R*)-6-hydroxy-5-methylramulosin (**11**), and (3*R*)-5-methylmellein (**12**) – were isolated from the endophytic fungal strain *Phomopsis* sp. XZ-01 of *Camptotheca acuminate*. Their structures were elucidated by spectroscopic analyses, including ^1^H- and ^13^C-NMR, 2D NMR (HSQC, HMBC, ^1^H-^1^H COSY and NOESY) and HR-FT-MS. Cytotoxic activities of these compounds were evaluated. Some of them showed weak selective activities.

## 1. Introduction

**Figure 1:**
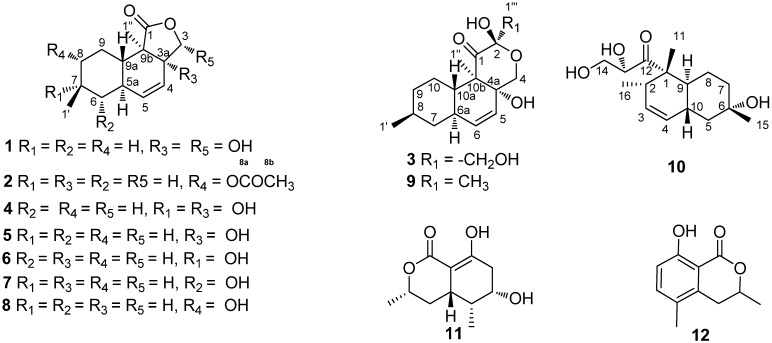
Structures of compounds **1-12**.

Endophytes, especially those found in medicinal plants, have drawn a lot of attention for the past few years as a rich and reliable source of bioactive and chemically novel compounds with huge medicinal and agricultural potential [1]. In the course of our exploration for bioactive or new chemical entities from the endophytic fungus of *Camptotheca acuminate* Decne (Cornaceae), numerous new compounds were obtained [2,3]. Continuous research on the secondary metabolisms of another endophytic fungus of *Camptotheca acuminate* (*Phomopsis* sp. XZ-01), led to the discovery of three new oblongolides C1 (**1**), P1 (**2**), and X1 (**3**), oblongolides B (**4**) [4], C (**5**) [4], D (**6**) [4], O (**7**) [3], P (**8**) [3] and U (**9**) [3], the new phomodiol 6-hydroxyphomodiol (**10**), (3*R*,4a*R*,5*S*,6*R*)-6-hydroxy-5-methyl-ramulosin (**11**) [5], and (3*R*)-5-methylmellein (**12**) [6]. In this paper, we report the isolation and structural elucidation of compounds **1-12** (Figure 1) and their anticancer activities.

## 2. Results and Discussion

**Figure 2:**
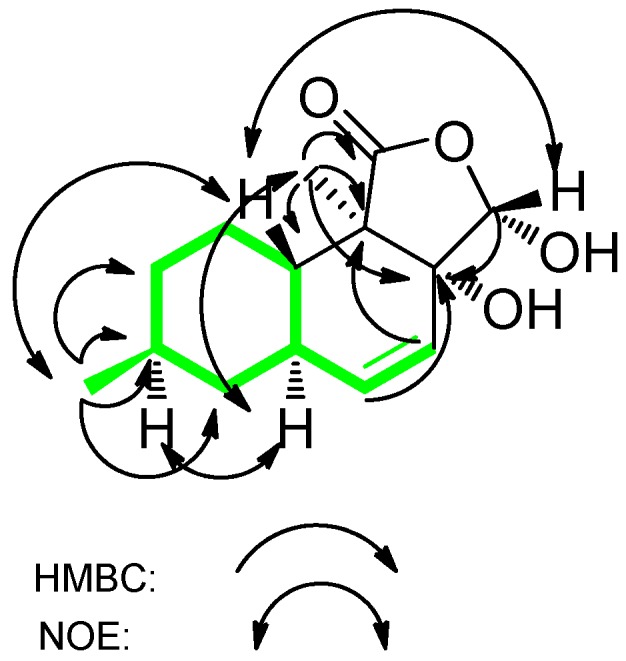
Key HMBC and NOE Correlations of compound **1**.

We obtained oblongolide C1 (**1**) as white needles and determined it to have the molecular formula C_14_H_20_O_4_ by HR-FT-MS. The ^13^C-NMR, DEPT and HSQC spectra of compound **1** showed 14 carbon signals: two methyl groups, three methylene groups, three methine groups, one hemiacetal methine (*δ*_C_ 100.6), a disubstituted olefin (*δ*_C_ 137.8 and 124.2), an oxygenated quaternary carbon (*δ*_C_ 78.7), a lactone carbonyl (*δ*_C_ 176.6), and a quaternary carbon. The ^1^H-^1^H COSY correlations between H-4 and H-5, H-5 and H-5a, H-5a and H-6, H-5a and H-9a, H-6 and H-7, H-7 and H-1′, H-8 and H-7, H-8 and H-9, H-9a and H-9 established the structure of a 9-carbon moiety (Figure 2, in green). Key HMBC correlations from H-1″ to C-1, C-3a, C-9a and C-9b, from H-5 to C-3a, from H-4 to C-9b, and from H-3 to C-3a established the planar structure of **1**. The relative configuration of **1** was deduced on the basis of NOESY spectroscopic data. The NOE correlations between H-7 and H-5a and between H-5a and H-1″ established the α-orientations of H-5a, H-7 and H-1″. NOESY cross-peaks from H-3 to H-9a and from H-9a to H-1′ indicated the β-orientations of H-3, H-9a and H-1′. A comparison of the ^1^H and ^13^C-NMR spectra of **1** with that of oblongolide C indicated that **1** was the 3α-hydroxy derivative of oblongolide C [4]. Therefore, we determined the structure of **1** to be 3α-hydroxyoblongolide C and it was named as oblongolide C1 for consistency with the literature [4]. 

Oblongolide P1 (**2**) was isolated as a white powder. The molecular formula C_16_H_22_O_4_ was deduced from HR-FT-MS and ^13^C-NMR. NMR data of **2** were similar to those of **1**, except that the hemiacetal methine [*δ*_H_ 5.69 (1H, d, *J* = 11.5 Hz) and *δ*_C_ 100.6, CH-3], quaternary carbon (*δ*_C_ 78.7, C-3a) and methylene [*δ*_H_ 1.82 (1H, m), *δ*_H_ 0.91 (1H, m) and *δ*_C_ 34.6, CH-8] in **1** were replaced by oxymethylene [*δ*_H_ 4.44 (1H, t, *J* = 8.6 Hz), *δ*_H_ 3.85 (1H, dd, *J* = 10.9, 8.9 Hz) and *δ*_C_ 70.1, CH_2_-3], methine [*δ*_H_ 2.78 (1H, m) and *δ*_C_ 44.6, CH-3a], oxymethine [*δ*_H_ 4.54 (1H, dt, *J* = 10.8, 4.4 Hz) and *δ*_C_ 77.1, CH-8], and there was an acetyl group in **2**. Key HMBC correlations from H-8 to C-8a, C-1′ and C-9a, from H-1′ to C-6, C-7 and C-8, from H-1″ to C-1, C-3a, C-9a and C-9b indicated the planar structure of **2**. We determined the relative configuration of **2** by analysis of the NOESY spectrum. The NOE correlations between H-8 and H-1′, between H-8 and H-9a, between H-8 and H-9β, and between H-1′ and H-6β established the β-orientations of H-1′, H-8 and H-9a. The NOE correlations between H-3a and H-1″, between H-3α and H-3a and between H-1″ and H-5a indicated the α-orientations of H-1″, H-3a and H-5a. A comparison of the ^1^H- and ^13^C-NMR data of **2** with those of oblongolide P [3] revealed that these two compounds had similar structures, except that an acetyl group was attached to the C-8 hydroxyl group in **2**. Therefore, we determined **2** to be 8-acetylobolngolide P and named it oblongolide P1.

**Table 1:** ^1^H- and ^13^C-NMR spectroscopic data of compounds **1** and **2** (**1** and **2** at 600 MHz, CDCl_3_, chemical shift values are in ppm relative to TMS; multiplicity and *J* values (in Hz) are presented in parentheses.

No.	1		2	
	δ_H_	δ_C_	δ_H_	δ_C_
1		176.6		179.4
3α			4.44 (t, 8.6)	70.1
3β	5.69 (d, 11.5)	100.6	3.85 (dd, 8.9, 10.9)	77.1
3a		78.7	2.78 (m)	44.6
4	5.53 (dd, 10.2, 2.8)	124.2	5.62 (dd, 12.8, 2.5)	122.2
5	5.79 (d, 9.9)	137.8	5.65 (d, 12.8)	133.0
5a	2.03 (m)	36.3	1.97 (m)	35.4
6α	0.84 (q, 12.4)	41.0	1.00 (m)	39.0
6β	1.90 (m)	41.0	1.94 (m)	39.0
7	1.50 (m)	32.7	1.68 (m)	37.4
8α	0.91 (m)	34.6		
8β	1.82 (m)	34.6	4.54 (dt, 4.4, 10.8)	77.1
9α	1.34 (dd, 12.4, 3.1)	25.6	1.38 (q, 12.2)	31.1
9β	1.79 (m)	25.6	2.10 (m)	31.1
9a	1.49 (m)	44.1	1.50 (m)	37.3
9b		51.5		42.8
1′	0.93 (d, 6.6)	22.2	0.94 (d, 6.5)	18.0
1″	1.18 (s)	9.5	1.16 (s)	16.1
8a				170.5
8b			2.06 (s)	21.1

Oblongolide X1 (**3**) was obtained as white oil. Its molecular formula, C_16_H_24_O_5_, was deduced on the basis of HR-FT-MS and ^13^C-NMR data. A comparison of the NMR data of **3** with those of known compound oblongolide X [7] indicated that **3** was a hydroxy-derivative of the latter. The HMBC correlations from H-1″′ to C-1 and C-2 located the hydroxyl substitution at C-2. The NOE correlations between H-10a and H-1′, between H-6a and H-8 and between H-6a and H-1″ determined the relative configuration of **3**. Therefore, we determined 3 to be 1″′-hydroxyoblongolide X and named it oblongolide X1.

**Table 2:** ^1^H- and ^13^C-NMR spectroscopic data of compound **3** (**3** at 600 MHz, CDCl_3_, chemical shift values are in ppm relative to TMS; multiplicity and *J* values (in Hz) are presented in parentheses.

No.	3	
	δ_H_	δ_C_
1	-	207.0
2	-	94.9
4α	3.57 (d, 12.4)	66.1
4β	4.63 (d, 12.4)	66.1
4a		78.2
5	5.36 (dd, 10.1, 2.8)	126.9
6	5.68 (dd, 10.1, 1.6)	136.2
6a	1.93 (m)	37.9
7α	1.86 (m)	41.1
7β	0.89 (m)	41.1
8	1.49 (m)	33.0
9α	1.77 (m)	34.8
9β	1.03 (m)	34.8
10α	1.26 (m)	26.8
10β	1.23 (m)	26.8
10a	2.33 (ddd, 11.5, 10.6 3.0)	43.8
10b		55.4
1′	0.93 (d, 6.5)	22.3
1‴	1.09 (s)	10.4
1‴	3.61 (d, 11.9)	65.2
1‴ β	3.95 (d, 11.9)	65.2

Compound **10** had the molecular formula C_16_H_26_O_4_, as established by HR-FT-MS and ^13^C-NMR spectra. ^1^H- and ^13^C-NMR data of **10** were similar to those of phomodiol [8], except that the methine signal [*δ*_H_ 1.46 (1H, m), CH-6] was replaced by a quaternary carbon (*δ*_C_ 70.0, C-6). Key HMBC correlations from H-15 to C-5, C-6 and C-7, from H-11 to C-1, C-2, C-9 and C-12, from H-16 to C-1, C-2 and C-3, from H-4 to C-2, C-5 and C-9 and from H-10 to C-3, C-6 and C-8 indicated the planar structure of **10**. The relative configuration of **10** was deduced on the basis of NOESY spectroscopic data. The NOE correlations between H-10 and H-11, between H-2 and H-11, between H-13 and H-11, between H-15 and H-10 and between H-9 and H-16 indicated β-orientation of the hydroxyl group (6-OH) and the α-orientation of the side chain attached to C-1. Therefore, the structure of **10** was determined. We named it 6-hydroxyphomodiol [8].

**Table 3:** ^1^H- and ^13^C-NMR spectroscopic data of compound **10** (600 MHz, in CDCl_3_, chemical shift values are in ppm relative to TMS; multiplicity and *J* values (in Hz) are presented in parentheses.

No.	10	
	δ_H_	δ_C_
1	–	5.15
2	2.18 (m)	39.5
3	5.58 (ddd, 9.9, 4.9, 2.6)	130.2
4	5.36 (d, 10.0)	129.0
5α	1.28 (m)	45.5
5β	1.75 (m)	45.5
6	–	70.0
7α	1.56 (dt, 13.6, 4.4)	39.4
7β	1.69 (dd, 14.1, 3.0)	39.4
8α	1.09 (brs)	22.8
8β	1.32 (m)	22.8
9	1.79 (m)	40.5
10	2.22 (m)	33.0
11	1.35 (s)	16.7
12	–	214.0
13	4.52 (brs)	75.7
14	4.03 (dd, 11.8, 3.6), 3.79 (dd, 11.7, 4.7)	63.3
15	1.27 (s)	31.6
16	0.84 (d, 7.0)	18.7

Besides the nine oblongolides, including three new ones, we isolated two more polyketides. We determined **11** to be (3*R*,4a*R*,5*S*,6*R*)-6-hydroxy-5-methylramulosin (**11**) [5] by a comparison of NMR data. This compound was previously isolated from a marine-derived fungus which was derived from the green alga *Codium fragile* [5]. The spectroscopic data of **12** were identical to those of the known compound (3*R*)-5-methylmellein, first isolated as the main phytotoxic metabolite of *Fusicoccum amygdale* [6].

### Cytotoxicity

The results of cytotoxic tests of compounds **1**-**12** are shown in Table 4. They exhibited no significant activity against the three tested cancer cell lines.

**Table 4:** Biological Activities of Compounds **1**-**12**.

Compound	Inhibitory rate(%)		
	HeLa	A549	HepG2
Oblongolide C1 (**1**)	-	-	18.01 ± 0.86
Oblongolide P1 (**2**)	-	-	28.59 ± 1.04
Oblongolide X1 (**3**)	-	-	27.89 ± 1.2
Oblongolide B (**4**)	-	-	-
Oblongolide C (**5**)	-	14.92 ± 0.86	-
Oblongolide D (**6**)	22.9 ± 0.78	13.82 ± 1.01	-
Oblongolide O (**7**)	-	-	-
Oblongolide P (**8**)	-	-	-
Oblongolide U (**9**)	-	18.76 ± 0.56	16.89 ± 1.01
6-Hydroxyphomodiol (**10**)	-	-	23.86 ± 1.21
(3*R*,4a*R*,5*S*,6*R*)-6-Hydroxy-5-methylramulosin (**11**)	-	-	-
(3*R*)-5-Methylmellein (**12**)	-	-	-

## 3. Experimental

### 3.1. General

Optical rotations were measured with a Perkin-Elmer 341 automatic polarimeter in methanol. IR spectra were recorded on a Nicolet AVATAR 330FT spectrometer. NMR spectra were taken on a Bruker Avance III-600 NMR spectrometer with TMS as an internal standard. HR-FT-MS data were acquired by using En Apex ultra 7.0 FT-MS. TLC was carried out using glass-precoated silica gel GF254 (Qingdao) and visualized under UV light or by spraying with vanillin (contains H_2_SO_4_) ethanol reagent. Sephadex LH-20 (40-70 µm, Amersham Pharmacia Biotech AB, Uppsala, Sweden), silica gel (200-300mesh, Qingdao Marine Chemical, Inc., Qingdao, China), and lichroprep reversed-phase RP-18 silica gel (40-63 µm, Merck, Darmstadt, Germany) were used for column chromatography (CC).

### 3.2. Fungal Material

The fungus (XZ-01) was isolated from current-year twigs (8-12 × 1-2 cm, length × diameter) of *Camptotheca acuminate* collected from the Jiangshi Natural Reserve, Shaowu, Fujian, China. It was identified as a non-sporulating fungus by traditional morphology. A BLAST search result showed that the internal transcribed spaces (ITS) sequence of XZ-01 was highly homologous (98% percent similarity) to that of a *Phomopsis* species (BCC 9789 [GU086404]), indicating that XZ-01 belongs to this genus.

### 3.3. Fermentation and Extraction

XZ-01 was cultivated on potato dextrose agar at 28 °C. The agar blocks were chopped and transferred into Erlenmeyer flasks (10 × 3 L), each containing 1 L of potato dextrose broth (PDB), and then fermented at 28 °C on a rotary shaker (150 rpm) for 7d. The culture was filtered to separate broth and mycelia. The culture broth was extracted with EtOAc (6 × 10 L) for six times. The combined organic layer was concentrated under vacuum to afford 3.2 g of residue.

### 3.4. Isolation and Spectral Data

The crude extract was separated into fifteen fractions (1-15) by column chromatography on RP-18 silica gel, eluted by methanol/H_2_O (0:100, 30:70, 50:50, 70:30, and 100:0). Fraction 3 (100 mg) was subjected to silica gel CC (step gradient, elution with 0-10% MeOH in CHCl_3_) to afford eleven fractions (3-1-3-11). Fractions 3-11 (4.9 mg) were further separated by silica gel CC (step gradient, elution with 22.2-33.3% EtOAc in hexane) to yield **4** (2.3 mg). Fraction 5 (92.1 mg) was separated by Sephadex LH-20 (elution with 100% methanol) to give three subfractions (fraction 5-1–5-3). Fraction 5-2 (23.6 mg) was purified by silica gel CC (step gradient, 7.7-50% EtOAc in hexane) to produce fraction 5-2-1. Fraction 5-2-1 (3.7 mg) was separated by silica gel (eluted with 50% CHCl_3_ in petroleum ether) to afford **11** (2mg). Fraction 6 (225.8 mg) was fractionated by Sephadex LH-20 CC (elution with 100% MeOH) to provide nine fractions (6-1–6-9). Fraction 6-1 (28.8 mg) was further purified by silica gel CC (step gradient, 0-17% MeOH in CHCl_3_) to furnish **6** (11.5 mg), **8** (2.6 mg) and **10** (6.4 mg). Fraction 7 (247.1 mg) was subjected to Sephadex LH-20 CC (elution with 100% MeOH) to give 5 fractions (7-1–7-5). Fraction 7-4 (36.1 mg) was purified by silica gel CC (elution with CHCl_3_) to yield **7** (3.1 mg). Fraction 10 (109 mg) was fractionated by Sephadex LH-20 CC (elution with 100% MeOH) to provide two fractions (10-1–10-2). Fraction 10-1 (72 mg) was further purified by silica gel CC (step gradient, elution with 0-10% MeOH in CHCl_3_) to give two subfractions (10-1-1 and 10-1-2). Fraction 10-1-2 (11.7 mg) was separated by silica gel CC (elution with 100% CHCl_3_) to yield **3** (3.8 mg). Fraction 11 (318.3 mg) was separated by Sephadex LH-20 (elution with 100% MeOH) to provide five fraction (11-1–11-5). Fraction 11-5 (23.9 mg) was further purified by silica gel CC (elution with 10% CHCl_3_ in petroleum ether) to afford **12** (22.8 mg). Fraction 11-3 (99 mg) was separated by silica gel CC (step gradient, elution with 0-10% MeOH in CHCl_3_) to give **5** (34 mg) and **9** (2.3 mg). Fraction 12 (117 mg) was fractionated by Sephadex LH-20 CC (elution with 100% MeOH) to provide three fractions (12-1–12-3). Fraction 12-1 (12.8 mg) was further separated by silica gel CC (elution with 33.3% CHCl_3_ in petroleum ether) to yield **2** (7.4 mg). Fraction 9 (232 mg) was separated by Sephadex LH-20 (elution with 100% MeOH) to give two fractions (9-1–9-2). Fraction 9-2 (38 mg) was purified by silica gel CC (step gradient, 0-12.3% MeOH in CHCl_3_) to yield **1** (5.7 mg). 

*Oblongolide C-1* (**1**): White needles; [α]*_D_*^20^: – 22.6 (c 0.0072, MeOH). IR (KBr) ν_max_ 2919, 2359, 1219, 772, 668 cm^–1^. ^1^H- and ^13^C-NMR: see Table 1; HR-FT-MS: m/z = 251.1281 [M − H]^-^ (calcd. for C_14_H_19_O_4_, 251.1283, Temperature: 180, Resolution: 125,508).

*Oblongolide O-1* (**2**): White powder; [α]*_D_*^20^: – 72.2(c 0.0025, MeOH). IR (KBr) ν_max_ 3344, 2922, 1588, 1383, 772 cm^–1^. ^1^H- and ^13^C-NMR: see Table 1; HR-FT-MS: m/z = 301.1418 [M + Na]^+^ (calcd. for C_16_H_22_O_4_Na, 301.1416, Temperature: 180, Resolution: 14,100).

*Oblongolide X-1* (**3**): White oil; [α]*_D_*^20^: – 21.7(c 0.0056, MeOH). IR (KBr) ν_max_ 3422, 1583, 773, 685 cm^–1^. ^1^H- and ^13^C-NMR: see Table 2; HR-FT-MS: m/z = 295.1541 [M − H]^-^ (calcd. for C_16_H_21_O_5_, 295.1545, Temperature: 180, Resolution: 106,466).

*6-Hydroxyphomodiol* (**10**): Transparent oil; [α]*_D_*^20^: + 43.3(c 0.002, MeOH). IR (KBr) ν_max_ 2365, 1223, 771 cm^–1^. ^1^H- and ^13^C-NMR: see Table 3; HR-FT-MS: m/z = 305.1736 [M + Na]^-^ (calcd. for C_16_H_26_NaO_4_, 305.1729, Temperature: 180, Resolution: 36,000).

### 3.5. Biological Assay

Cancer cell lines were derived from the cell bank of The Chinese Academy of Sciences. Cells were seeded at a density of 5 × 10^3^/100 µL medium in 96-well microtiter plate and treated with the compounds at the concentration of 20 µg/mL. Viable cells were incubated with MTT (5 mg/mL) for 4 h and formazan precipitate was dissolved in 100 µL DMSO and the absorbance at 490 nm was measured by Multimode Detector DTX880 (Beckman Coulter).

## 4. Conclusions

Four new compounds, oblongolides C1 (**1**), P1 (**2**), X1 (**3**), 6-hydroxyphomodiol (**10**), together with eight known compounds were isolated from the endophytic fungus *Phomopsis* sp. XZ-01. oblongolides C1 (**1**), P1 (**2**), X1 (**3**), and 6-hydroxyphomodiol (**10**) showed modest selective activities against HepG2 cancer cell lines. Oblongolide C (**5**) exhibited minor selective activity against A549. 
